# Novel clinically meaningful scores for the ICIQ-UI-SF and ICIQ-FLUTS questionnaires in women with stress incontinence

**DOI:** 10.1007/s00192-023-05657-4

**Published:** 2023-10-11

**Authors:** Shamima Islam Nipa, David Cooper, Alyaa Mostafa, Suzanne Hagen, Mohamed Abdel-Fattah

**Affiliations:** 1 Department of Rehabilitation Science, Bangladesh Health Professions Institute (BHPI), Savar, Bangladesh; 2 Health Services Research Unit, Institute of Applied Health Sciences, University of Aberdeen, Aberdeen, UK; 3 Aberdeen Centre for Women’s Health Research, Institute of Applied Health Sciences School of Medicine, Medical Sciences and Nutrition, University of Aberdeen, Aberdeen, UK; 4 Glasgow Caledonian University, Cowcaddens Rd., Glasgow, UK; 5 Aberdeen Centre for Women’s Health Research, School Medicine, Medical Sciences and Nutrition, University of Aberdeen, Aberdeen, UK

**Keywords:** Clinically important difference, ICIQ-SF, ICIQ-FLUTS, Minimal important difference, PGI-I, Stress urinary incontinence

## Abstract

**Aim:**

To establish, for the first time, the clinically important differences for the ICIQ-UI-SF and ICIQ-FLUTS questionnaires following surgical and conservative treatments for stress-predominant urinary incontinence in women.

**Methods:**

Data from the SIMS and OPAL randomised controlled trials were analysed using an anchor-based method. Clinically important difference (CID; score change indicating a successful outcome) and minimal important difference (MID; score change indicating the smallest noticeable difference) were estimated using the PGI-I scale as the anchor.

**Results:**

For ICIQ-UI-SF, following surgical management, CIDs were 5.0 (95%CI 4.3, 5.6) at 1 year and 4.9 points (95%CI 4.2, 5.5) at 3 years, while following conservative management, CIDs were 4.0 (95%CI 3.4, 4.5) at 1 year and 4.6 points (95%CI 4.0, 5.2) at 2 years. For ICIQ-FLUTS, the CID was 3.4 points (95%CI 2.9, 4.0) at 1 year for both surgical and conservative management. MIDs for ICIQ-UI-SF, after surgical treatment, were 4.7 (95% CI 3.2, 6.1) at 1 year and 1.6 points (95%CI –0.2, 3.0) at 3 years, and after conservative treatment they were 1.7 (95% CI 1.0, 2.5) at 1 year and 1.9 points (95%CI 1.1, 2.7) at 2 years. For ICIQ-FLUTS, MIDs were 1.8 (95% CI 0.6, 3.1) at 1 year and 3.2 points (95%CI 2.0, 4.4) at 2 years after surgical treatment, and 1.3 (95%CI 0.6, 1.9) at 1 year and 1.9 points (95%CI 1.1, 2.6) at 2 years after conservative treatment.

**Conclusion:**

Our study is the first to establish the CID for the ICIQ-UI-SF and ICIQ-FLUTS that women would associate with a successful outcome 3-years post-surgery and 2-years post-conservative treatment of stress-predominant urinary incontinence. The MID was lower following conservative compared to surgical treatment.

## Introduction

Urinary incontinence (UI) is a debilitating condition that affects the patient’s quality of life (QoL). Patient-reported outcome measures (PROMs) are now recognised as the most relevant outcomes to assess following treatment of UI [1, 2]. However, it is well-recognised that the assessment of interventions for UI should be multidimensional and hence also include objective measures such as cough-stress and pad tests [3].

There are a number of validated PROMs currently used in this field, including International Consultation on Incontinence Questionnaire – Urinary Incontinence Short Form (ICIQ-UI-SF) [4], International Consultation on Incontinence Questionnaire – Female Lower Urinary Tract Symptoms (ICIQ-FLUTS) [5] and Patient Global Impression of Improvement (PGI-I) [6].

**Fig. 1:**
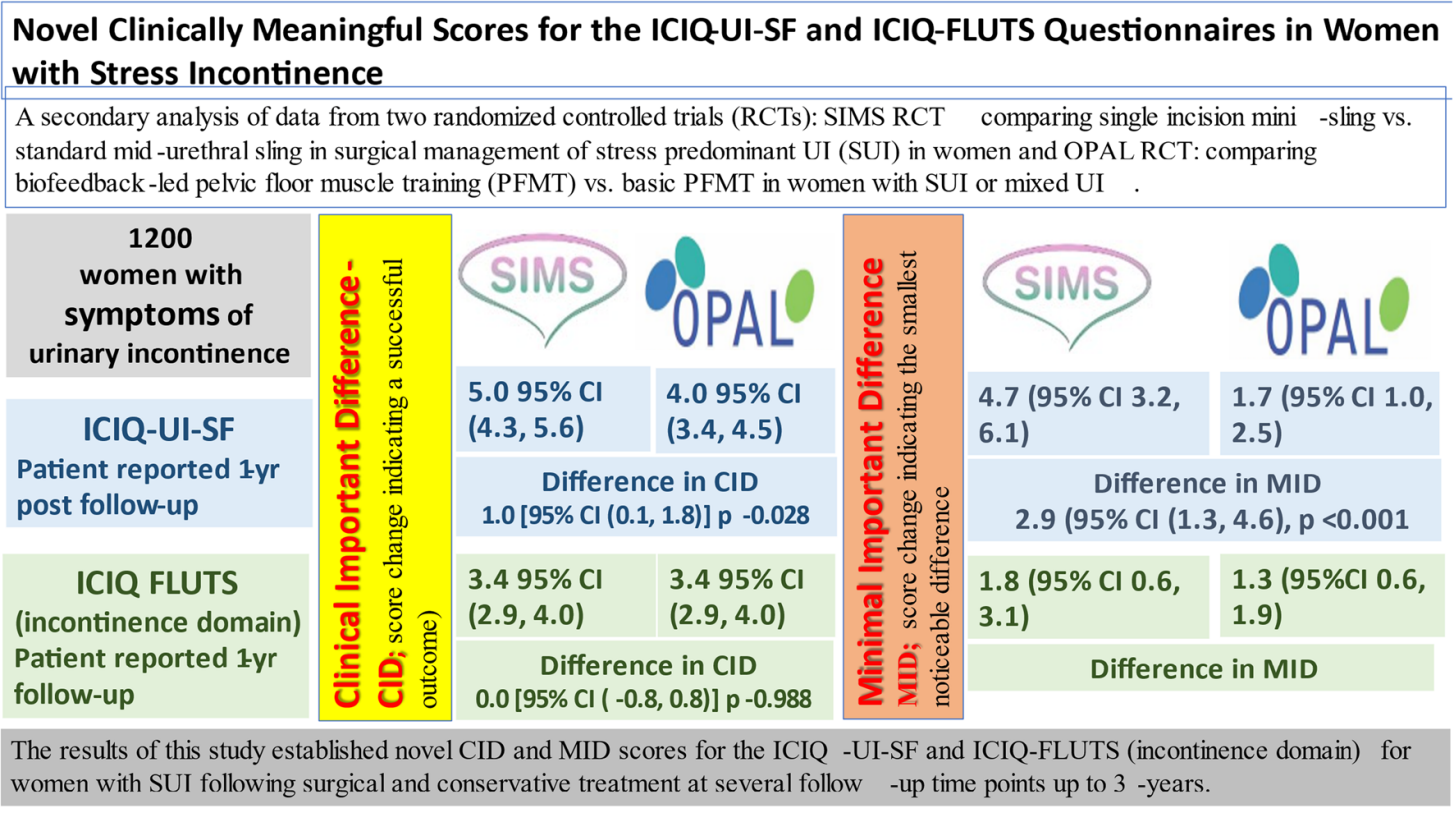
Infographic summary

The ICIQ-UI-SF is a validated patient-reported measure of symptom severity and impact of UI on the individual’s QoL [4]. It is short and simple; hence, it is frequently used in research and clinical practice. The ICIQ-FLUTS is a longer questionnaire that provides a comprehensive picture for the severity of all domains of urinary symptoms (not limited to UI); however, due to its length, it is used mainly for detailed assessment in specialist centres and in clinical trials (Fig. 1).

There are no ‘norm’ values for ICIQ-UI-SF or ICIQ-FLUTS questionnaires; they are used to assess the impact of treatment by calculating the difference between post and pre-intervention scores. Hence, it is important to determine what constitutes meaningful clinically important differences from the patient’s perspective [7]. This is essential to guide clinicians when counselling patients about what to expect following surgical and conservative treatment. This information is also most useful for researchers when planning treatment trials and for comparing results of clinical trials.

Previous research attempted to make clinical sense of symptom severity questionnaire score changes by testing for statistically significant differences. However, these may have little clinical relevance to the patients and were influenced by the relatively small sample sizes of the studies [8].

Minimal clinically important difference (MCID) has been described previously as the smallest statistically significant difference in questionnaire score which patients perceive as beneficial [9–11]. However, MCID did not differentiate between mean score change that represents an ‘improvement’ compared to those changes that would represent a ‘successful outcome’. Furthermore, they did not differentiate between surgical and conservative treatments.

We, therefore, hypothesise that two separate measures are needed: clinically important difference (CID) as the change in a PROM score that patients considered is truly worthwhile (i.e. a successful outcome), and minimum important difference (MID), the score change that represents the ‘smallest noticeable improvement’ [12, 13].

In this study we aim to establish, for the first time, the CID and the MID for both ICIQ-UI-SF and ICIQ-FLUTS at different follow-up time points for women undergoing surgical and conservative treatment for UI.

## Materials and methods

This is a secondary analysis of data from two randomised controlled trials (RCTs). SIMS RCT: comparing single incision mini-sling vs. standard mid-urethral sling in surgical management of stress predominant UI (SUI) in women with follow-up to 3-years [14] and OPAL RCT: comparing biofeedback-led pelvic floor muscle training (PFMT) vs. basic PFMT in women with SUI or mixed UI, with follow-up to 2-years [15]. In both RCTs patient-reported outcome measures were chosen as the primary outcome. Ethics approval, including data sharing statements, was granted for both SIMS and OPAL RCTs from the North and the West of Scotland Research Ethics Committees, respectively.

All RCTs’ participants completed validated symptom severity and QoL questionnaires pre-treatment, including ICIQ-UI-SF [4] and ICIQ-FLUTS [5]. The same questionnaires were completed at different follow-up time alongside the PGI-I [6] questionnaire. The CID and MID were estimated using the SIMS data at 3, 6, 15, 24 and 36 months for outcomes following surgical management and the OPAL data at 6, 12 and 24 months for outcomes following conservative management. We compared CID and MID estimated for surgical versus conservative management at 1- and 2-years follow-up.

### The ICIQ-UI-SF

Is a relatively short questionnaire, formed of six items; two demographic items and three items for rating of symptoms in the preceding 4-weeks (frequency of UI episodes, the amount of leakage, and overall impact of UI). The total score of these three items gives the ICIQ-UI-SF score with range 0 to 21 points, where higher scores indicate greater symptom severity and higher impact on the women’s QoL [4]. The sixth item is the self-diagnostic question for type of UI and is not included in the final score.

### The ICIQ-FLUTS

Is the full-length questionnaire and formed of 12 items divided into three domains of incontinence which are scored separately (5 items and scoring 0–20); voiding (3 items scoring 0–12); and filling (4 items scoring 0–16); and sexual function (2 items) and QoL (5 items) [5]. All items have simple additive scores, where a higher score indicates greater symptom severity. Unlike in the ICIQ-UI-SF, the bothersome score is not included when calculating the ICIQ-FLUTS domain scores, i.e. the domain scores do not include the impact on women’s QoL. There is no total score calculated for the full ICIQ-FLUTS. The incontinence domain is the most relevant to patients reporting on the outcome of UI treatment; hence, this will be used in our analyses.

### The PGI-I

Is a global index to rate the response to treatment. A transition scale asks participants to what extent their condition has improved since receiving treatment, includes ‘very much better/much better/a little better/no change/a little worse/much worse/very much worse’. Typically, these responses are analysed using ordered logistic regression. It has been validated as a PROM measure for assessing the overall patient assessment for the outcome of treatment [6]. Over the past 2 decades, many clinical trials in the field have used the PGI-I as a patient reported outcome and invariably defining success as ‘very much/ much better’ [16–21].

## Data analysis

We used an anchor-based method to estimate the CID and MID for the ICIQ UI-SF and ICIQ-FLUTS (incontinence domain) questionnaires, with PGI-I being the anchor variable.

### The clinically important difference (CID)

To indicate success of the intervention, the anchor was constructed from the PGI-I by assuming that those responding ‘much improved’ or ‘very much improved’ were a treatment success, and those choosing the other five responses were treatment failure/unsuccessful. All participants were thus categorised as either success or unsuccessful/ failure to determine the CID.

### The minimal important difference (MID)

Was the value for which the participant perceived a small improvement in their UI post-intervention. This was obtained by comparing the group of participants responding ‘improved’, classed as success, to the PGI-I to the group of participants responding ‘same’, classed as failure. Participants responding ‘much improved’, ‘very much improved’, ‘worse’, ‘much worse’ and ‘very much worse’ were not included in this analysis.

The CID and MID were estimated by comparing groups defined by change on the anchor and participants were included if they had a baseline and at least one follow-up measurement for the relevant outcome. The ICIQ-UI-SF and ICIQ-FLUTS are on the scales 0 to 21 and 0 to 20, respectively, and in the simplest case the comparison between the change and no change groups to estimate the CID and MID could be done using a two-sample t-test. Both the SIMS and OPAL trials made repeated measures on participants and to make full use of the data, by adjusting for the different timepoints when outcomes were measured at more than one observation on each participant, repeated measures mixed effects linear regression analysis was conducted. The dependent variables were either change in ICIQ-UI-SF or ICIQ-FLUTS (incontinence domain). Fixed effects were the categorical variable corresponding to success on the anchor variable, as the coefficient on this binary variable can be interpreted as the difference between the groups. Fixed effects for time point and an interaction between time point and change on the anchor variable were also included. A random intercept was included for participant to adjust for repeated measures. The estimate of the important differences was a linear combination of the interaction and the variable indicating a change on the anchor variable. All statistical analyses were performed with Stata Statistics 14 software *(StataCorp. 2015. Stata Statistical Software: Release 14. College Station, TX*, *StataCorp LP.).*

The comparisons of the CID and MID between surgical versus conservative management have been made using an independent two sample t-test.

## Results

In the SIMS RCT analysis, 600 women were included [14]. Both the ICIQ-UI-SF and PGI-I were completed by 70% and 64% at 1- and 3-years while ICIQ-FLUTS (incontinence domain) and PGI-I were completed by 76% (1-year) and 68% (3-years), respectively. Women were aged from 26–87 years old; the mean age was 51 years (SD 11) and the median body mass index was 28 kg/m^2^ (IQR 25.0, 32.1).

The OPAL RCT randomised 600 women [15]. At 1 and 2-years’ post-treatment 83% and 76% of women completed both the ICIQ-UI-SF and PGI-I, while the ICIQ-FLUTS (incontinence domain) and PGI-I were completed by 62% and 55%, respectively. Women were aged from 20–83 years old; the mean age was 47 years (SD 11.5), and the median body mass index was 27.1 kg/m2 (IQR 24.0, 32.2).

Table 1 shows the number of observations where the PGI-I and the change from baseline on the outcomes ICIQ-UI-SF and ICIQ-FLUTS incontinence domain were available, summarising the data available for estimating the CID and MID of the two outcomes. There are a greater number of observations available for SIMS compared to OPAL for the ICIQ-UI-SF, but the opposite is the case for the ICIQ-FLUTS (incontinence domain). The larger SD indicates more variation in the SIMS data compared to OPAL, and for both variables, the largest increases in score and therefore worsening on both outcomes are observed for the OPAL data. The mean change in the ICIQ-UI-SF score in the SIMS trial at 3-years was 9.9 (SD 5.5) and in the OPAL trial at 2-years was 3.4 (SD 4.5) (Table 1). For the ICIQ-FLUTS incontinence domain, the mean change in score in the SIMS RCT at 3-years follow-up was 3.5 (SD 3.5) and in the OPAL RCT at 2-years follow-up was 1.1 (SD 2.8) (Table 1).

**Table 1:** The mean changes of the ICIQ-UI-SF & ICIQ-FLUTS for SIMS and OPAL

	Trials	Observations	Mean	SD	Min	Max
ICIQ-UI-SF	SIMS	1586	–9.9	5.5	–21	9
	OPAL	1364	–3.4	4.5	–18	18
ICIQ-FLUTS	SIMS	235	–3.5	3.5	–14	5
	OPAL	514	–1.1	2.8	–10	8

### CID

Table 2 shows the CID estimates for the ICIQ-UI-SF and ICIQ-FLUTS (incontinence domain) for both RCTs at all different follow-up time points.

- CID for the ICIQ-UI-SF, the CID estimates were 5.0 (95% CI 4.3, 5.6) and 4.8 (95% CI 4.1, 5.5) points improvement at 1- and 2-years follow-up, respectively, for women having surgical management (Table 2). The CID estimates were 4.0 (95% CI 3.4, 4.5) and 4.6 (95% CI 4.0, 5.2) points improvement at 1- and 2-years follow-up, respectively, for women having conservative management (Table 2). Comparing the CID estimates following surgical and conservative management, there were significant differences at 1-year (mean difference 1.0, 95% (CI 0.1, 1.9); *p* = 0.028) but not at 2-years (mean difference 0.2, 95% CI (–0.7, 1.1); *p* = 0.720).
- CID for the ICIQ-FLUTS incontinence domain, the CID estimate was 3.4 (95% CI 2.9, 4.0) for women having both surgical and conservative management of UI at 1-year follow-up (Table 2).

**Table 2:** The clinical important difference (CID) for ICIQ-UI-SF and ICIQ-FLUTS for SIMS and OPAL

Time-points	SIMS trial data (95% CI)	OPAL trial data (95% CI)	SIMS CID v OPAL CID Difference (95% CI); p-value
CID with ICIQ-UI-SF			
3 months	–5.6 (–6.3, –4.9)	n/a	n/a
6 months	n/a	–4.2 (–4.8, –3.7)	n/a
1 year^1^	–5.0 (–5.6, –4.3)	–4.0 (–4.5, –3,4)	–1.0(–1.8, –0.1); 0.028
2 years	–4.8 (–5.5, –4.1)	–4.6 (–5.2, –4.0)	–0.2(–1.1, 0.7); 0.720
3 years	–4.9 (–5.5, –4.2)	n/a	n/a
CID with ICIQ-FLUTS (Incontinence Score)			
6 months	n/a	–3.2(–3.8, –2.7)	n/a
1 year^1^	–3.4(–4.0, –2.9)	–3.4(–4.0, –2.9)	0.0(–0.8, 0.8);0.988
2 years	–3.6(–4.1, –3.0)	–3.9(–4.5, –3.4)	0.4(–0.4, 1.2); 0.371
3 years	–3.4(–3.9, –2.9)	n/a	n/a

^1^The 1-year outcome refers to the 12 month follow-up in OPAL and the 15 month follow-up in SIMS

### MID

Table 3 shows the MID estimates for the ICIQ-UI-SF and ICIQ-FLUTS (incontinence domain) for both RCTs at all different follow-up time points.

- For ICIQ-UI-SF, the MID was 4.7 (95% CI (3.2, 6.1)) and 3.0 (95% CI (1.6, 4.5)) points improvement at 1- and 2-years follow-up, respectively, following surgical treatment. While the MID following conservative management for women with UI was 1.7 (95%CI (1.0, 2.5) and 1.9 (95%CI (1.1, 2.7)) points improvement at 1- and 2-years, respectively. As was the case with the CID there is a significant difference between the 1-year estimates of the MID mean difference 2.9 (95% CI (1.3, 4.6), *p* < 0.001).
- For the ICIQ-FLUTS (incontinence domain): the MID was 1.8 (95%CI (0.6, 3.1)); and 3.2 (95%CI 2.0, 4.4) points improvement at 1- and 2-years follow-up, respectively, following surgical treatment. While the MID following conservative management for women with UI was 1.3 (95%CI (0.6, 1.9)) and 1.9 (95% CI (1.1, 2.6)) points improvement at 1- and 2-years follow-up, respectively.

**Table 3:** Minimum important difference (MID) for both SIMS and OPAL trial data (ICIQ-UI-SF) and (ICIQ-FLUTS)

Time-points	SIMS MID (95% CI)	OPAL MID (95% CI)	SIMS MID v OPAL MID Difference (95% CI); p-value
MID with ICIQ-UI-SF			
3 months	–3.8(–5.5, –2.0)	n/a	n/a
6 months	n/a	–2.0(–2.7, –1.2)	n/a
1 year^1^	–4.7(–6.1, –3.2)	–1.7(–2.5, –1.0)	–2.9(–4.6, –1.3); < 0.001
2 years	–3.0(–4.5, –1.6)	–1.9(–2.7, –1.1)	–1.1(–2.8, 0.5); 0.165
3 years	–1.6(–3.0, –0.2)	n/a	n/a
MID with ICIQ-FLUTS (Incontinence score)			
6 months	n/a	–1.4(–2.1, –0.7)	n/a
1 year	–1.8(–3.1, –0.6)	–1.3(–1.9, –0.6)	–0.6(–2.0, 0.8); 0.424
2 years	–3.2(–4.4, –2.0)	–1.9(–2.6, –1.1)	–1.3(–2.8, 0.1); 0.068
3 years	–0.7(–1.8,0.5)	n/a	n/a

^1^The 1-year outcome refers to the 12 month follow-up in OPAL and the 15 month follow-up in SIMS

## Discussion

This study determined estimates of the CIDs and MIDs of two PROMs, most widely used in clinical practice and clinical trials, for women with UI in different treatment pathways [22]. Our results are the first to estimate the CID in the ICIQ-UI-SF at different time-points up to 3-years follow-up and the only study to differentiate between CID/MID scores following surgical and conservative treatment of SUI in women. Following surgical treatment, score improvements of 5.6 and 4.9 points at 3 months and 3 years indicate women reporting a ‘successful outcome’. While 3.5 points improvement represent the CID in ICIQ-FLUTS (incontinence domain) scores at both the 1 and 3 years. Following conservative treatment, our study showed that score improvement of 4.2 and 4.6 points represents the CID on the ICIQ-UI-SF, while score improvement of 3.2 and 3.9 points represents the CID on the ICIQ-FLUTS (incontinence domain) at 6-month and 2-year follow-up, respectively. The estimated CIDs on both questionnaires are of significant clinical relevance to both clinicians and the patients as they indicate patient-reported successful outcome following surgical and conservative interventions. CID at short- and longer-term follow-up are important for clinical trials planning when ‘success’ is required to be the primary outcome rather than improvement such as surgical trials. These are most valuable in estimation of sample size and as treatment outcome measures.

Our results are the first to estimate the MID in the ICIQ-UI-SF: improvement in the score of 4.7 and 3.0 points at 1- and 2-years after surgery, respectively, indicate the smallest improvement in UI that participants perceived to be important. While 1.7 and 1.9 points improvement at 1- and 2-years follow–up, respectively, indicate the smallest improvement in UI participants perceive following conservative management. This shows that women have higher expectations from surgical intervention as indicated by greater improvement in their symptom severity scores being reported as the smallest noticeable improvement. Having MID estimates specific to conservative treatment of UI is important in designing clinical trials in this field as they can be more clinically relevant and may reduce sample size and consequently research waste when clinical ‘improvement’ is preferred as the primary outcome.

Our results suggest women undergoing conservative treatment have MID to be almost 50% of their CID in contrast for women undergoing surgical treatment who seem to have higher expectation from their intervention. In the latter women rate/expect their MID to be close to CID.

Sirls et al. [12] reported minimal CID (95% CI) in surgical management comparing retropubic vs trans-obturator mid-urethral slings in women with SUI, based on the PGI-I, as –4.8 (95% CI –5.6, –3.9) at 12 months and –4.2 (–5.1, –3.4) at 24 months, respectively. These results are in agreement with our study results where CID were 5.0 (95% CI (4.3, 5.6)) and 4.8 (95% CI 4.1, 5.5)) at 1-year and 2-years follow-up, respectively, following surgical intervention.

The current literature of minimal CID following conservative management for women with SUI is less clear. One RCT by Nystrom et al. [13] compared two different formats of PFMT administrated either online or by mail. They reported a lower minimal CID of 2.5 points on ICIQ-UI-SF at 4-month follow-up compared to our CID for ICIQ-UI-SF of 4.2 points improvement at 6 months after similar conservative management [13]. Lim et al. [22] evaluated the efficacy of pulsed magnetic stimulation for conservative management of UI in women. Their study estimated that the minimal CID was 3.8 points for the ICIQ-UI SF at 12-month follow-up, which is closer to our estimated CID. The most likely explanation in these differences is that in Nystrom et al., they included women responses of ‘very much better, much better and a little better’ while Lim et al.’s study used a similar definition of success to our CID, i.e. women’s responses of ‘very much better, much better’ only. The differences in definition in the above two studies [13, 22] supports our hypothesis for the need to differentiate between the MID and the CID.

The ICIQ-FLUTS is a detailed and comprehensive assessment of the patients’ urinary symptoms (storage, voiding and continence domains) and not confined to UI. Our results are novel and the first to detect the CID and the MID of the incontinence domain score in this questionnaire. There are no studies in the literature to compare our results.

The results from our study indicate that MID for ICIQ-UI-SF and ICIQ-FLUTS (incontinence domain) seem to fall over the follow-up duration following surgical management for UI; however, the CID was stable. This may indicate that women’s perception of small change/ ‘improvement’ may change over time with changes in priority to their general health. The Cochrane review on mid-urethral slings also concluded that the success rate of all surgical treatment for SUI tends to decline with time [23]. In contrast, the results for the CID and MID had slightly risen over the follow-up following conservative management. This is best explained by the difference in the management strategies. Women are likely to expect an immediate and significant improvement in UI symptoms following surgery, while in conservative management, women may believe more in the concept of gradual improvement over time.

We used PGI-I as the anchor for this analysis. The PGI-I is a global index that is widely used to rate the response of a condition to a therapy (transition scale). A simple, direct and easy to use single-item scale is intuitively understandable to clinicians and patients [24]. The PGI-I has excellent construct validity compared to various assessment variables: incontinence episode frequency, the Incontinence Quality of Life Questionnaire, and fixed-volume stress pad test [25]. In summary, PGI-I provides a robust validated and more global review of the treatment outcome and more encompassing of the range of benefits and potential harms [26]; hence, it is best suited for this analysis to show the CID and MID on both questionnaires.

In our study we used the anchor-based method that depends on women reported outcome using a single measure for women with UI perspective [27, 28] (PGI-I). This global rating of change considers more information regarding QoL than other clinical tools. The Food Drug Administration (FDA) guidelines [28] recommended the use of anchor-based methods regarding PRO measures, which depends on personal experiences and observations only for responder data.

Our study has several strengths, being the first to report CID and MID for ICIQ-UI-SF and ICIQ-FLUTS (incontinence domain) at different follow-up time points and up-to 2 and 3-years for both conservative and surgical treatment for SUI in women, respectively. Other strengths include the use of data from multi-centre prospective RCTs and utilising the methodology anchor-based methods. Our results are directly relevant in the clinical setting for counseling women before and after interventions for SUI and to enable proper sample size calculations in clinical trials. It is important to reiterate that the novel CID and MID established for ICIQ-UI-SF and ICIQ-FLUTS should be utilised as one part of multidimensional assessment of outcomes following SUI interventions which will also include objective measures and impact on women’s QOL and sexual function. There are a number of limitations; we used data collected for women with stress predominant UI from two RCTs. As a result, the findings do not apply to other types of UI. It is also important to keep in mind that CID and MID estimation can differ amongst populations [17]. Women’s expectations may differ between developed and developing countries, and these may affect the results. The inherited lack of consideration of factors such as age, co-morbid conditions and socio-economic impact when calculating the patient reported questionnaire scores may impact the CID and MID estimates.

## Conclusion

The results of this study established novel CID and MID scores for the ICIQ-UI-SF and ICIQ-FLUTS (incontinence domain) for women with SUI following surgical and conservative treatment at several follow-up time points up to 3 years. These novel scores are most valuable in planning clinical trials for estimation of sample size and during post-treatment assessment as part of multi-dimensional outcome measures. In the clinical setting, they will help counselling of women regarding potential treatment outcomes.

## Data Availability

The study data will be shared in accordance with the ‘National Institute for Health Research position on the sharing of research data’. All requests for access to the data should be directed to the CI and will be managed by the University of Aberdeen in accordance with the NIHR position statement. Release of data will be subject to a data use agreement with the third party requesting the data.
